# Association of vitamin E and coenzyme Q10 pretreatment with embryological and pregnancy outcomes in women with diminished ovarian reserve: a retrospective study

**DOI:** 10.3389/fendo.2026.1936380

**Published:** 2026-10-21

**Authors:** Shiyu Wang, Tianjuan Wang, Wenxiang Zhang, Yajing Liu, Yunxia Cao

**Affiliations:** 1 Department of Obstetrics and Gynecology, Reproductive Medicine Center, the First Affiliated Hospital of Anhui Medical University, Hefei, Anhui, China; 2 National Health Commission Key Laboratory of Study on Abnormal Gametes and Reproductive Tract, Hefei, Anhui, China; 3 Engineering Research Center of Biopreservation and Artificial Organs, Ministry of Education, Hefei, Anhui, China

**Keywords:** coenzyme Q10, diminished ovarian reserve, *in vitro* fertilization/intracytoplasmic sperm injection, pregnancy outcomes, top-quality embryos, transferable embryos, vitamin E

## Abstract

**Objective:**

To evaluate the associations of adjunctive vitamin E (Vit E) and coenzyme Q10 (CoQ10) supplementation with embryological and pregnancy outcomes in patients with diminished ovarian reserve (DOR) undergoing IVF/ICSI-ET.

**Methods:**

In this retrospective observational study, 949 patients with DOR undergoing IVF/ICSI-ET were included. According to adjunctive treatment before controlled ovarian stimulation, patients were categorized into the Control (n=338), Vit E+CoQ10 (n=260), CoQ10 (n=224), or Vit E (n=127) groups. Demographic characteristics, ovarian reserve indicators, stimulation parameters, embryological outcomes, and pregnancy outcomes were compared. Multivariable Poisson regression models with a log link and robust standard errors adjusted for demographic characteristics, ovarian reserve indicators, stimulation parameters, fertilization method, and treatment cycle initiation year were used to assess associations between supplementation regimens and embryological outcomes. A 2 × 2 exposure-based interaction model including Vit E use, CoQ10 use, and their interaction was applied.

**Results:**

Baseline characteristics and stimulation parameters did not differ significantly among groups. The distribution of treatment cycle initiation year also did not differ significantly among the groups (P = 0.325). The numbers of transferable and top-quality embryos differed significantly across groups and were highest in the Vit E+CoQ10 group. Pairwise comparisons showed that Vit E+CoQ10 was associated with more transferable embryos than Control and Vit E, while the CoQ10 group had more transferable embryos than Control and Vit E. The Vit E+CoQ10 group also had more top-quality embryos than the other groups. After multivariable adjustment, combined supplementation and CoQ10 supplementation were associated with increased numbers of transferable embryos, whereas combined supplementation did not differ significantly from CoQ10 alone. A positive statistical interaction between Vit E and CoQ10 was observed for the number of top-quality embryos. Clinical pregnancy, live birth, and early miscarriage rates did not differ significantly.

**Conclusions:**

In patients with DOR undergoing IVF/ICSI-ET, CoQ10 supplementation was associated with a higher transferable-embryo count, whereas combined Vit E+CoQ10 supplementation was associated with a higher top-quality-embryo count. However, these embryological differences were not associated with improved pregnancy outcomes.

## Introduction

Diminished ovarian reserve (DOR) refers to a condition in women of reproductive age in which the number of recruitable follicles or oocytes in the ovaries is lower than expected for women of the same age. Clinically, it is commonly assessed using a combination of indicators, including anti-Müllerian hormone (AMH), antral follicle count (AFC), basal follicle-stimulating hormone (FSH), age, and previous ovarian response to stimulation (1). This condition suggests reduced female reproductive potential and may be associated with a decreased chance of conception. Owing to a shortened reproductive window and reduced likelihood of natural conception, patients often face greater pressure in fertility decision-making, and some therefore choose *in vitro* fertilization and embryo transfer treatment. However, patients with DOR often respond poorly to controlled ovarian stimulation, which may affect the number of oocytes retrieved, the number of available embryos, and subsequent pregnancy outcomes. At present, there is no effective method to reverse DOR. Clinical management therefore emphasizes early fertility planning, individualized ovarian stimulation protocols, and strategies to maximize the use of limited oocytes. When necessary, assisted reproductive technology or fertility preservation strategies may be adopted as part of a comprehensive approach. In addition, adjuvant treatment strategies such as growth hormone, testosterone, dehydroepiandrosterone, coenzyme Q10 (CoQ10), and vitamin E (Vit E) have been explored to improve ovarian response and assisted reproductive outcomes, but the evidence regarding their efficacy remains inconsistent (2). CoQ10 and Vit E have mainly been studied as antioxidant adjuvant therapies, which is consistent with the mechanistic hypothesis that oxidative stress and mitochondrial dysfunction may contribute to ovarian functional decline (3). Moreover, as oral agents, both are convenient to use, are associated with relatively few common adverse effects, and are therefore readily accepted by patients and widely used in clinical practice.

Among existing studies, the evidence for CoQ10 is relatively more concentrated. Some studies have suggested that it may improve ovarian stimulation response and embryological parameters in women with DOR, although evidence regarding live birth outcomes remains insufficient (4). Mechanistically, CoQ10 primarily participates in mitochondrial energy metabolism and exerts antioxidant effects, whereas Vit E mainly protects cellular structures through lipid membrane antioxidant activity (5, 6). Thus, the two agents may theoretically have complementary effects. However, clinical studies on the combined use of CoQ10 and Vit E as adjuvant treatment in patients with DOR remain limited, and their potential benefits require further validation. Based on retrospective data from 949 patients with DOR who underwent *in vitro* fertilization/intracytoplasmic sperm injection-embryo transfer (IVF/ICSI-ET) at the Reproductive Medicine Center of our hospital, this study grouped patients according to their oral use of CoQ10 and Vit E, including combined use, single-agent use, and non-use. We analyzed the associations of combined supplementation with ovarian stimulation response, embryological parameters, and clinical pregnancy outcomes, aiming to provide real-world evidence for adjuvant treatment in patients with DOR.

## Methods

### Patient population

In this retrospective observational study, a total of 949 patients with DOR who underwent IVF/ICSI-ET treatment at the Reproductive Medicine Center of the First Affiliated Hospital of Anhui Medical University between January 2018 and December 2023 were included. Only the first IVF/ICSI-ET treatment cycle for each patient during the study period was included, and all subsequent treatment cycles were excluded. Thus, each patient contributed only one treatment cycle to the analysis. This study was approved by the Ethics Committee of the First Affiliated Hospital of Anhui Medical University (No. PJ-2021-09-08). At the time of routine clinical treatment, all patients provided written informed consent stating that their clinical data might be used for clinical scientific research. For this specific retrospective analysis, the requirement for additional study-specific written informed consent was waived by the Ethics Committee. All data were handled in accordance with institutional requirements for patient privacy and confidentiality. At our center, DOR was diagnosed when the total AFC in both ovaries was ≤5 and at least one of the following criteria was met: a serum AMH concentration of ≤1.2 ng/mL or a basal FSH concentration of ≥10 IU/L (1). AFC was assessed by transvaginal ultrasonography, and basal FSH was measured during the early follicular phase. Patients subsequently entered the IVF/ICSI-ET treatment cycle. Individualized controlled ovarian stimulation protocols were then developed according to patient age, ovarian reserve, and previous treatment history (7). When follicular development met the criteria for final oocyte maturation, oocyte retrieval was performed, followed by fertilization using either IVF or ICSI. The resulting embryos were cultured *in vitro*, and suitable embryos were selected for transfer based on embryonic developmental stage, morphological grading, and endometrial conditions (8). Day-3 cleavage-stage embryos were evaluated according to blastomere number, degree of fragmentation, and blastomere symmetry using the embryo scoring system routinely applied at our center. Embryos with more than four blastomeres and a total score of ≥5 were considered transferable, whereas embryos with a score of 9–10 were classified as top-quality. Blastocysts cultured to days 5–7 were graded according to the Gardner grading system. Blastocysts with both the inner cell mass and trophectoderm grades of C or higher were considered transferable. Day-5 blastocysts graded ≥3BB and day-6 or day-7 blastocysts graded ≥4BB were classified as top-quality embryos. Pregnancy outcomes were continuously followed up and recorded. Clinical pregnancy was defined as the ultrasonographic visualization of an intrauterine gestational sac 30 days after embryo transfer. Early miscarriage was defined as pregnancy loss before 12 weeks of gestation. Live birth was defined as the delivery of at least one live-born infant at or after 28 weeks of gestation. Exclusion criteria were as follows: (1) concomitant medical conditions that may adversely affect pregnancy outcomes, including uterine malformations, severe endometrial pathologies, or other serious chronic diseases; (2) male-factor infertility resulting in failure to achieve fertilization; (3) chromosomal abnormalities or carrier status for hereditary diseases; (4) patients using other adjunctive therapies that help improve ovarian function, such as growth hormone and dehydroepiandrosterone; (5) cycle cancellation or interruption before embryological outcomes could be adequately evaluated; or (6) missing essential baseline, supplementation, ovarian stimulation, or embryological outcome data required for the primary analyses. Patients who did not undergo embryo transfer during the study observation period were retained in the baseline, ovarian stimulation, and embryological outcome analyses when the corresponding data were complete. Patients with missing pregnancy outcome data were retained in these analyses but were excluded only from the corresponding analyses of clinical pregnancy, live birth, and early miscarriage, which were conducted using available cases. For patients receiving CoQ10 and/or Vit E, the supplementation regimens were based on previous reports: CoQ10 was administered at a dose of 200 mg three times daily for 2 months, and Vit E was administered at a dose of 100 mg three times daily for 2 months before the initiation of controlled ovarian stimulation (4, 9). Supplementation was recommended at the discretion of the treating physician, based on the physician’s clinical judgment and interpretation of the available evidence, and was initiated only when accepted by the patient; treatment was not randomly assigned. Patients were classified into the Control, Vit E+CoQ10, CoQ10, and Vit E groups according to their documented supplement use before controlled ovarian stimulation. The Control group was subject to the same eligibility criteria as the supplementation groups and comprised patients with DOR who had no documented use of either Vit E or CoQ10 before controlled ovarian stimulation. Completion of the 2-month supplementation regimen was assessed using medication information documented in the medical records and confirmed directly with the patients. The patient selection process and availability of embryo-transfer and pregnancy outcome data are shown in **Figure 1**.

**Figure 1:**
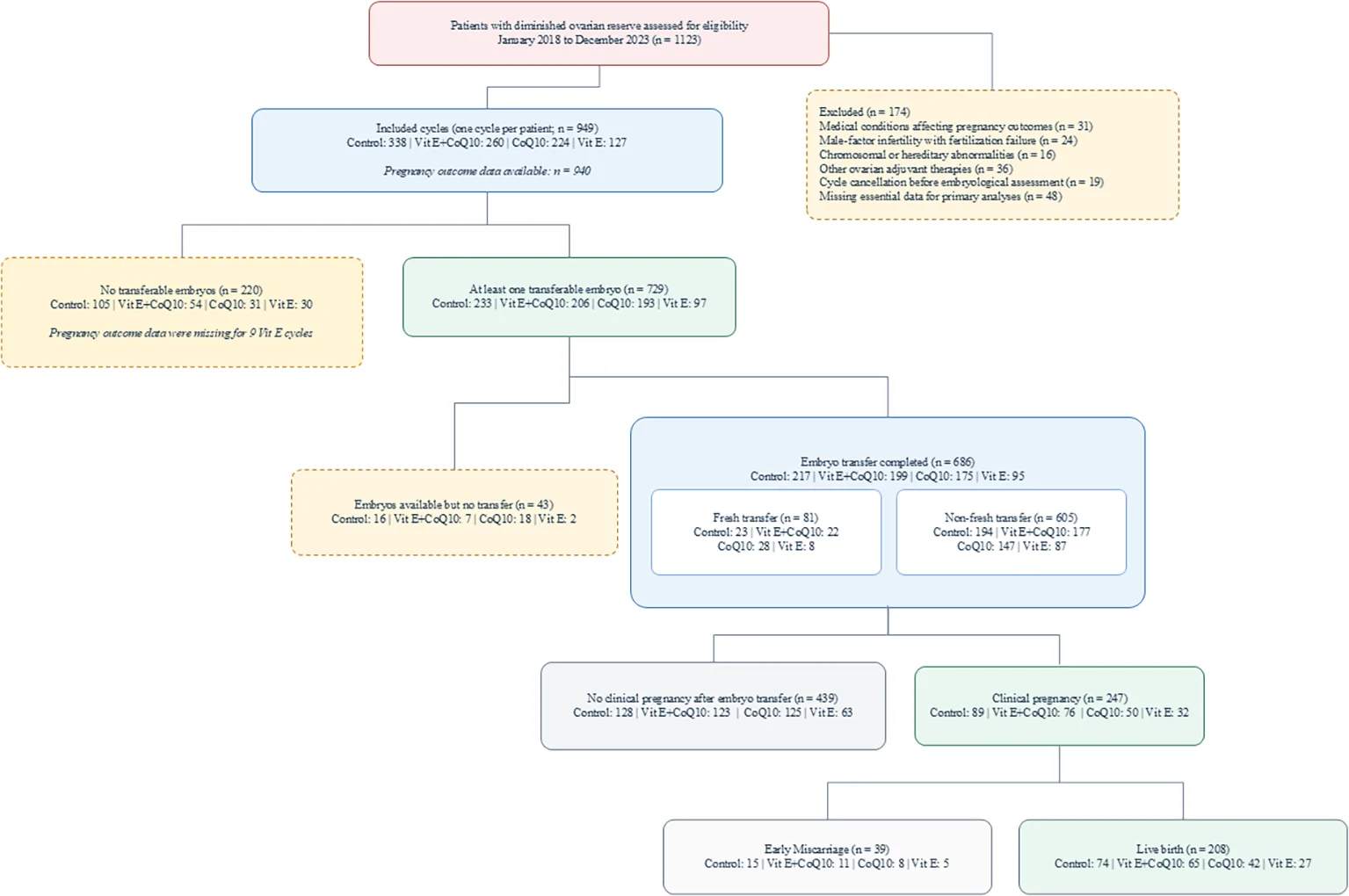
Patient selection and outcome availability. The flow diagram presents the initial cohort, exclusions, final treatment groups, availability of transferable embryos, embryo-transfer status, and pregnancy outcomes during the study observation period.

Clinical data were collected from the medical records, including demographic characteristics, ovarian reserve indicators, controlled ovarian stimulation parameters, embryological outcomes, and pregnancy outcomes of the first embryo transfer. Specifically, the collected variables included the year of treatment cycle initiation, patient age, husband’s age, body mass index (BMI), infertility duration, primary infertility, basal FSH, AFC, AMH, basal estradiol (E2), gonadotropin-releasing hormone (GnRH) antagonist protocol, total gonadotropin dosage, duration of gonadotropin stimulation, fertilization method, number of oocytes retrieved, number of metaphase II oocytes, number of two-pronuclear (2PN) embryos, number of transferable embryos, number of top-quality embryos, day of embryo transfer, number of embryos transferred, fresh embryo transfer, clinical pregnancy, live birth, and early miscarriage. Given the retrospective design and the evaluation of multiple embryological and clinical outcomes, these analyses were considered exploratory, and no single primary embryological outcome was prespecified.

### Statistical analysis

The participants were divided into four groups according to the use of CoQ10 and Vit E: the Control group, which received neither CoQ10 nor Vit E; the Vit E+CoQ10 group, which received both Vit E and CoQ10; the CoQ10 group, which received CoQ10 alone; and the Vit E group, which received Vit E alone. Categorical variables were presented as frequencies and percentages. Continuous variables were expressed as mean ± standard deviation (SD) for normally distributed data and as median with interquartile range (IQR) for non-normally distributed data. For comparisons among the four groups, normally distributed continuous variables were analyzed using one-way analysis of variance (ANOVA), while non-normally distributed continuous variables were analyzed using the Kruskal-Wallis H test. Categorical variables were compared using the chi-square test or Fisher’s exact test, as appropriate. For the assessment of cycle cancellation rates, the denominator comprised 968 otherwise eligible cycles, including the 949 cycles included in the main analysis and the 19 cycles cancelled or interrupted before embryological assessment. Clinical pregnancy, live birth, and early miscarriage were evaluated per initiated treatment cycle among patients with documented pregnancy outcome data. Accordingly, early miscarriage represented the proportion of initiated treatment cycles resulting in early miscarriage rather than the conditional proportion of clinical pregnancies ending in early miscarriage.

Variables showing statistically significant differences in the overall comparison among the four groups were further subjected to *post hoc* pairwise comparisons. For normally distributed continuous variables, pairwise comparisons were performed using the Bonferroni method after one-way analysis of variance. For non-normally distributed continuous variables, a significant Kruskal–Wallis test was followed by Dunn’s *post hoc* test with Holm adjustment for multiple pairwise comparisons. For categorical variables, pairwise comparisons were performed using the chi-square test or Fisher’s exact test, with Bonferroni correction for multiple comparisons.

Multivariable Poisson regression models were considered the primary analyses of the associations between treatment regimens and embryological outcomes, whereas the non-parametric pairwise comparisons were treated as unadjusted supplementary analyses. For count outcome variables, such as the number of transferable embryos and top-quality embryos, generalized linear models with a Poisson distribution and log link were fitted. Huber–White robust standard errors were used to account for possible misspecification of the conditional variance. Incidence rate ratios (IRRs) and their 95% confidence intervals (CIs) were calculated, and Wald χ² tests were used to assess statistical significance. The multivariable models were adjusted for clinically selected potential confounders, including female age, partner age, body mass index, infertility duration, primary infertility status, basal FSH, AFC, AMH, basal estradiol, GnRH antagonist protocol use, total gonadotropin dosage, duration of gonadotropin stimulation, fertilization method, and calendar year of treatment cycle initiation. Calendar year was entered as a categorical variable, with 2018 as the reference category. Model dispersion was evaluated using the Pearson χ² statistic and deviance divided by their residual degrees of freedom, with values substantially greater than 1 indicating overdispersion. Potential excess zeros were assessed by comparing the observed and model-expected proportions of zero counts and by fitting zero-inflated Poisson models as sensitivity analyses. Adjusted marginal mean counts and their 95% CIs for each supplementation group were obtained by averaging model-based predictions over the observed covariate distribution. Meanwhile, based on a 2 × 2 exposure-based interaction model, Vit E use and CoQ10 use were included in the model as two binary factors, together with their interaction term, to estimate the conditional association of Vit E, the conditional association of CoQ10, and the Vit E × CoQ10 statistical interaction.

All statistical analyses were performed using IBM SPSS Statistics version 22.0 (IBM Corp., Armonk, NY, USA), and a two-sided P value < 0.05 was considered statistically significant.

## Results

A total of 949 patients with DOR were included in this study, comprising 338 in the Control group, 260 in the Vit E+CoQ10 group, 224 in the CoQ10 group, and 127 in the Vit E group. Among the 968 cycles included in the cancellation-rate assessment, 19 were cancelled or interrupted before embryological outcomes could be adequately evaluated. According to the documented supplementation categories, cancellations occurred in 2/340 cycles (0.59%) in the Control group, 8/268 cycles (2.99%) in the Vit E+CoQ10 group, 5/229 cycles (2.18%) in the CoQ10 group, and 4/131 cycles (3.05%) in the Vit E group. Cancellation rates did not differ significantly among the four groups (Fisher–Freeman–Halton exact P = 0.072). The measured baseline characteristics did not differ significantly among the four groups. Of the 949 included treatment cycles, 686 proceeded to embryo transfer during the study observation period, whereas 263 did not. Among the 263 cycles without embryo transfer, 220 yielded no transferable embryos. The remaining 43 cycles yielded at least one transferable embryo that was cryopreserved, but no frozen–thawed embryo transfer was performed within the study observation period. Pregnancy outcome data were available for 940 of the 949 treatment cycles. For nine cycles, all in the Vit E group, no transferable embryos were obtained and no embryo transfer was performed, while the pregnancy outcome fields were not documented. To avoid imputing unrecorded outcomes, these nine cycles were treated as missing in the analyses of clinical pregnancy, live birth, and early miscarriage but were retained in the baseline and embryological outcome analyses. The distribution of treatment cycle initiation year also did not differ significantly among the four groups (χ² = 16.896, df = 15, P = 0.325). There were no statistically significant differences among the groups in patient age, husband’s age, BMI, duration of infertility, proportion of primary infertility, basal FSH, AFC, AMH, basal E2 levels, use of GnRH antagonist protocol, total gonadotropin dosage, or duration of ovarian stimulation (all *P* > 0.05). Similarly, no significant differences were observed in fertilization method (IVF or ICSI), number of oocytes retrieved, number of metaphase II (MII) oocytes, number of 2PN embryos, day of embryo transfer, number of embryos transferred, or the proportion of fresh embryo transfers (all *P* > 0.05). However, statistically significant differences were found among the four groups in the number of transferable embryos and the number of top-quality embryos (both *P* < 0.001). The Vit E+CoQ10 group had higher numbers of transferable embryos and top-quality embryos compared with the other groups. In terms of pregnancy outcomes, no statistically significant differences were observed among the four groups in clinical pregnancy rate, live birth rate, or early miscarriage rate (all *P* > 0.05) (**Table 1**). Among the 247 cycles resulting in clinical pregnancy, 39 ended in early miscarriage, corresponding to a conditional early miscarriage proportion of 15.789%.

**Table 1:** Comparison of baseline characteristics and reproductive outcomes among the overall population and four treatment groups.

Variables	Total population(n=949)	Control group(n=338)	Vit E+CoQ10 group(n=260)	CoQ10 group (n=224)	Vit E group (n=127)	H/χ^2^	P
Year of treatment cycle initiation (n [%])						16.896	0.325
2018	95/949 (10.011%)	32/338 (9.467%)	25/260 (9.615%)	16/224 (7.143%)	22/127 (17.323%)		
2019	87/949 (9.168%)	30/338 (8.876%)	26/260 (10.000%)	19/224 (8.482%)	12/127 (9.449%)		
2020	121/949 (12.750%)	40/338 (11.834%)	29/260 (11.154%)	36/224 (16.071%)	16/127 (12.598%)		
2021	216/949 (22.761%)	77/338 (22.781%)	54/260 (20.769%)	58/224 (25.893%)	27/127 (21.260%)		
2022	202/949 (21.286%)	76/338 (22.485%)	60/260 (23.077%)	40/224 (17.857%)	26/127 (20.472%)		
2023	228/949 (24.025%)	83/338 (24.556%)	66/260 (25.385%)	55/224 (24.554%)	24/127 (18.898%)		
Age of patient (years, median [IQR])	37.000 (32.000, 41.000)	37.000 (32.000, 41.750)	36.000 (33.000, 41.000)	38.000 (34.000, 40.000)	36.000 (30.000, 42.000)	4.455	0.216
Age of patient’s husband (years, median [IQR])	36.000 (32.000, 41.000)	37.000 (31.250, 42.000)	36.000 (32.000, 40.000)	37.000 (33.000, 40.250)	36.000 (30.000, 44.000)	1.100	0.777
BMI (kg/m², median [IQR])	22.500 (20.600, 24.600)	22.700 (20.800, 24.775)	22.500 (20.675, 24.400)	22.300 (20.500, 24.150)	23.030 (19.830, 25.240)	2.466	0.481
Infertility duration (years, median [IQR])	2.000 (1.000, 4.000)	2.000 (1.000, 3.000)	2.000 (1.000, 3.000)	3.000 (1.000, 4.000)	2.000 (2.000, 4.000)	5.975	0.113
Primary infertility (n/N [%])	390/949 (41.096%)	136/338 (40.237%)	102/260 (39.231%)	93/224 (41.518%)	59/127 (46.457%)	2.001	0.572
FSH (IU/L, median [IQR])	9.980 (7.580, 14.200)	9.940 (7.490, 13.200)	9.450 (7.922, 12.768)	11.167 (7.461, 16.151)	10.960 (6.895, 17.310)	3.885	0.274
AFC (n, median [IQR])	3.000 (2.000, 4.000)	3.000 (2.000, 4.000)	3.000 (2.000, 4.000)	3.000 (2.000, 4.000)	3.000 (2.000, 4.000)	3.727	0.292
AMH (ng/mL, median [IQR])	0.530 (0.340, 0.760)	0.530 (0.323, 0.770)	0.490 (0.360, 0.740)	0.565 (0.360, 0.790)	0.530 (0.385, 0.710)	1.174	0.759
Estradiol, E2 (pg/mL, median [IQR])	138.100 (94.270, 178.230)	140.450 (101.560, 174.250)	139.500 (98.950, 180.105)	135.000 (82.000, 188.740)	135.060 (82.600, 173.760)	4.308	0.230
GnRH antagonist protocol (n/N [%])	433/949 (45.627%)	151/338 (44.675%)	107/260 (41.154%)	116/224 (51.786%)	59/127 (46.457%)	5.681	0.128
Total gonadotropin dosage (IU, median [IQR])	2600.000 (2008.000, 3166.000)	2600.000 (2006.250, 3100.000)	2650.000 (2025.000, 3200.000)	2593.000 (2016.250, 3169.000)	2561.000 (1976.000, 3192.500)	0.309	0.958
Gn stimulation duration (days, median [IQR])	9.000 (8.000, 11.000)	9.000 (8.000, 11.000)	9.000 (8.000, 10.000)	9.000 (7.000, 11.000)	10.000 (8.000, 11.000)	7.580	0.056
IVF fertilization method (n/N [%])	827/949 (87.144%)	305/338 (90.237%)	223/260 (85.769%)	192/224 (85.714%)	107/127 (84.252%)	4.681	0.197
Total oocytes retrieved (n, median [IQR])	2.000 (1.000, 3.000)	2.000 (1.000, 3.000)	2.000 (1.000, 3.000)	2.000 (1.000, 3.000)	2.000 (1.000, 3.000)	3.249	0.355
Metaphase II oocytes (n, median [IQR])	1.000 (1.000, 2.000)	1.000 (1.000, 2.000)	1.500 (1.000, 2.000)	1.000 (1.000, 2.000)	1.000 (1.000, 2.000)	4.581	0.205
2PN cleavage embryos (n, median [IQR])	1.000 (1.000, 2.000)	1.000 (1.000, 2.000)	1.000 (1.000, 2.000)	1.000 (1.000, 1.000)	1.000 (1.000, 1.500)	3.333	0.343
Transferable embryos (n, median [IQR])	1.000 (1.000, 1.000)	1.000 (0.000, 1.000)	1.000 (1.000, 2.000)	1.000 (1.000, 1.000)	1.000 (1.000, 1.000)	18.560	<0.001
Top-quality embryos (n, median [IQR])	0.000 (0.000, 1.000)	0.000 (0.000, 1.000)	1.000 (0.000, 1.000)	0.000 (0.000, 1.000)	0.000 (0.000, 1.000)	42.750	<0.001
Day of embryo transfer (day, median [IQR])	5.000 (5.000, 5.000)	5.000 (5.000, 5.000)	5.000 (5.000, 5.000)	5.000 (5.000, 5.000)	5.000 (5.000, 5.000)	2.264	0.520
Transferred embryos (n, median [IQR])	1.000 (1.000, 1.000)	1.000 (1.000, 1.000)	1.000 (1.000, 1.000)	1.000 (1.000, 1.000)	1.000 (1.000, 1.000)	4.312	0.230
Fresh transfer (n/N [%])	81/686 (11.808%)	23/217 (10.599%)	22/199 (11.055%)	28/175 (16.000%)	8/95 (8.421%)	4.413	0.220
Clinical pregnancy (n/N [%])	247/940 (26.277%)	89/338 (26.331%)	76/260 (29.231%)	50/224 (22.321%)	32/118 (27.119%)	3.024	0.388
Live birth (n/N [%])	208/940 (22.128%)	74/338 (21.893%)	65/260 (25.000%)	42/224 (18.750%)	27/118 (22.881%)	2.778	0.427
Early miscarriage (n/N [%])	39/940 (4.149%)	15/338 (4.438%)	11/260 (4.231%)	8/224 (3.571%)	5/118 (4.237%)	0.266	0.966

Categorical variables are presented as n/N (%), where N represents the number of patients with available data for the corresponding outcome. Fresh embryo transfer, embryo culture duration, and the number of transferred embryos were evaluated among the 686 patients who underwent embryo transfer during the study observation period, including 217 in the Control group, 199 in the Vit E+CoQ10 group, 175 in the CoQ10 group, and 95 in the Vit E group. Clinical pregnancy, live birth, and early miscarriage were evaluated per initiated treatment cycle among the 940 patients with documented pregnancy outcome data, including 338, 260, 224, and 118 patients in the respective groups. Early miscarriage therefore represents the proportion of initiated treatment cycles resulting in early miscarriage.

Variables showing significant differences in the overall comparison (number of transferable embryos and number of top-quality embryos) were further analyzed with pairwise comparisons between groups. For the number of transferable embryos, the Vit E+CoQ10 group had significantly higher counts than the Control group (*P* = 0.003) and the Vit E group (*P* = 0.011). The CoQ10 group had significantly higher counts than the Control group (P = 0.025) and the Vit E group (P = 0.038), whereas no statistically significant difference was observed between the Vit E+CoQ10 and CoQ10 groups (**Figure 2**). For the number of top-quality embryos, the Vit E+CoQ10 group showed significantly higher counts than the Control group, the CoQ10 group, and the Vit E group (all *P* < 0.001) (**Figure 3**). These unadjusted comparisons describe the observed group differences, whereas primary inference was based on the multivariable-adjusted Poisson regression analyses presented below.

**Figure 2:**
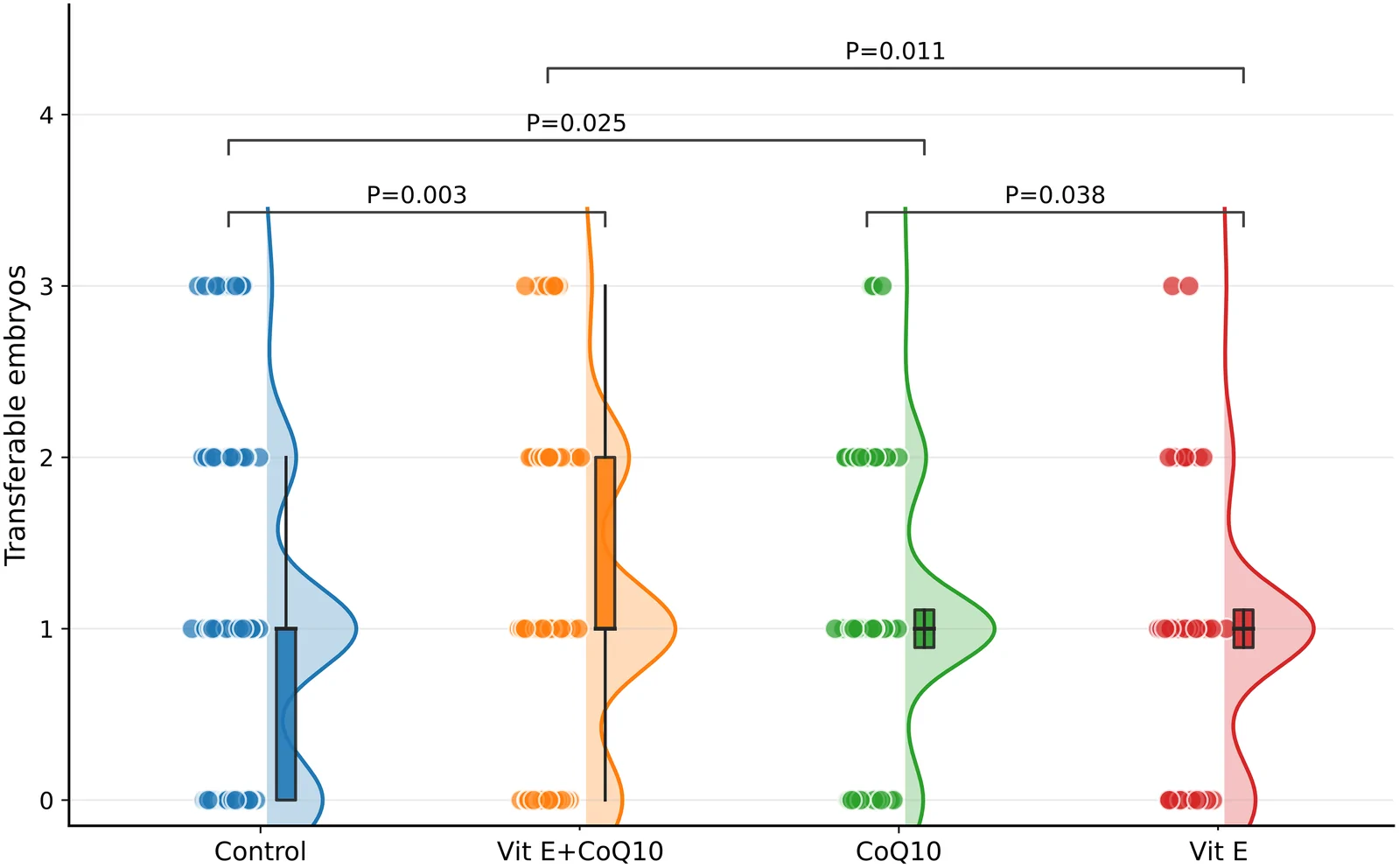
Comparison of the number of transferable embryos among the four groups. Data distributions and medians (interquartile ranges) are presented. The figure presents unadjusted comparisons among the four supplementation groups. The overall difference was assessed using the Kruskal–Wallis test, followed by Dunn’s *post hoc* test with Holm adjustment for multiple pairwise comparisons. Holm-adjusted P values for statistically significant comparisons are shown.

**Figure 3:**
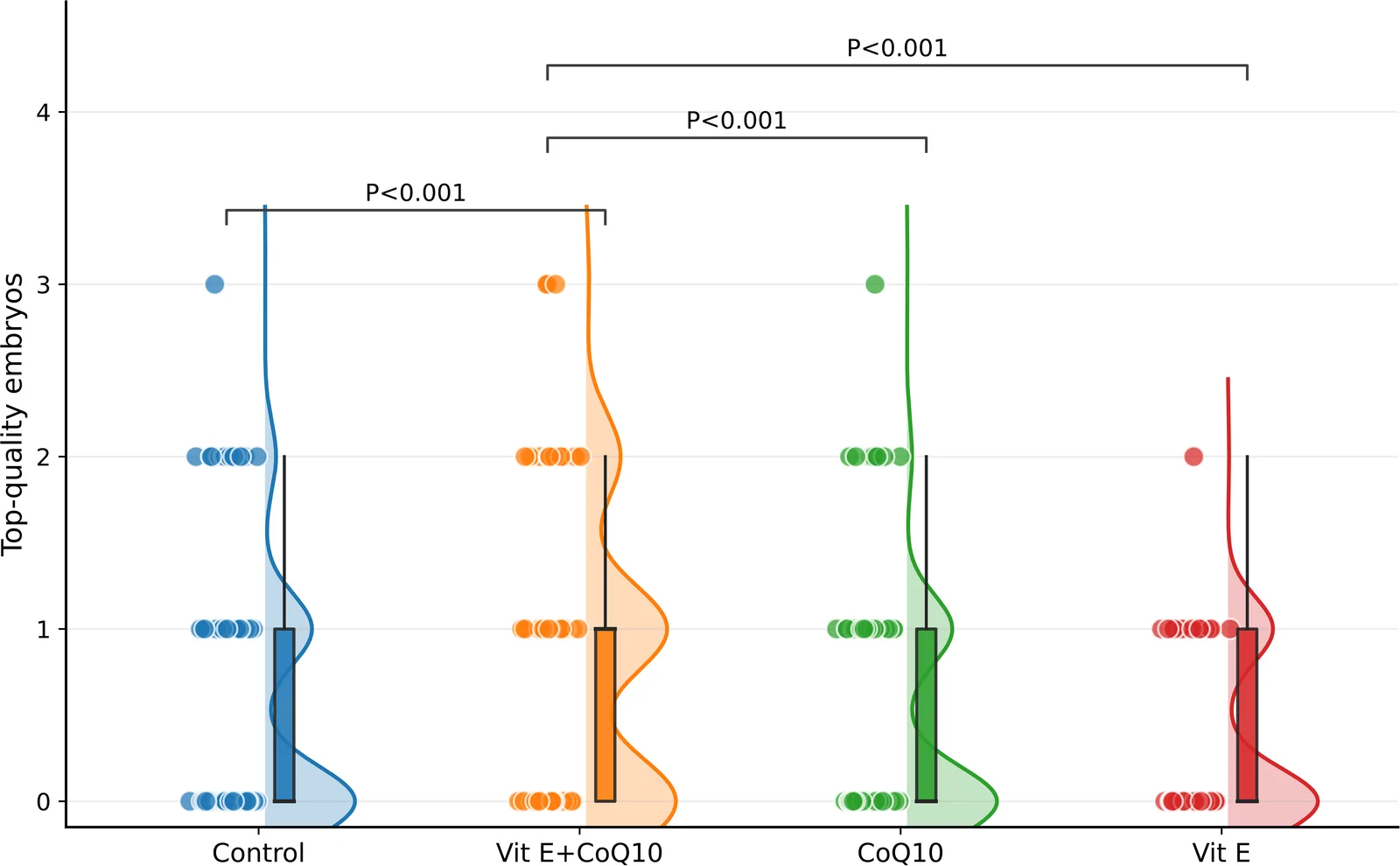
Comparison of the number of top-quality embryos among the four groups. Data distributions and medians (interquartile ranges) are presented. The figure presents unadjusted comparisons among the four supplementation groups. The overall difference was assessed using the Kruskal–Wallis test, followed by Dunn’s *post hoc* test with Holm adjustment for multiple pairwise comparisons. Holm-adjusted P values for statistically significant comparisons are shown.

A multivariable generalized linear regression model was used to further examine the associations between treatment regimen and embryological outcomes after adjustment for the specified covariates and treatment cycle initiation year. For the number of transferable embryos, compared with the Control group, the Vit E+CoQ10 group was associated with a significantly higher expected count (IRR = 1.214, 95% CI: 1.076–1.368, *P* = 0.002), as was the CoQ10 group (IRR = 1.148, 95% CI: 1.022–1.289, *P* = 0.020), whereas the difference in the Vit E group was not statistically significant (*P* = 0.671). The 2 × 2 exposure-based interaction analysis showed a significant positive association of CoQ10 with the number of transferable embryos (IRR = 1.148, 95% CI: 1.022–1.289, *P* = 0.020), while Vit E and the Vit E × CoQ10 statistical interaction were not statistically significant (both *P* > 0.05). For the number of top-quality embryos, the Vit E+CoQ10 group had significantly higher counts than the Control group (IRR = 1.578, 95% CI: 1.307–1.906, *P* < 0.001), the CoQ10 group (IRR = 1.858, 95% CI: 1.483–2.328, *P* < 0.001), and the Vit E group (IRR = 2.158, 95% CI: 1.638–2.843, *P* < 0.001). In the 2 × 2 exposure-based interaction model, Vit E use was associated with a lower expected number of top-quality embryos when CoQ10 was absent (IRR = 0.731, 95% CI: 0.550–0.972, P = 0.031), whereas the conditional association of CoQ10 use when Vit E was absent was not statistically significant (IRR = 0.849, 95% CI: 0.671–1.075, P = 0.175). The Vit E × CoQ10 statistical interaction was positive and statistically significant (IRR = 2.541, 95% CI: 1.767–3.654, P < 0.001) (**Figure 4**).

**Figure 4:**
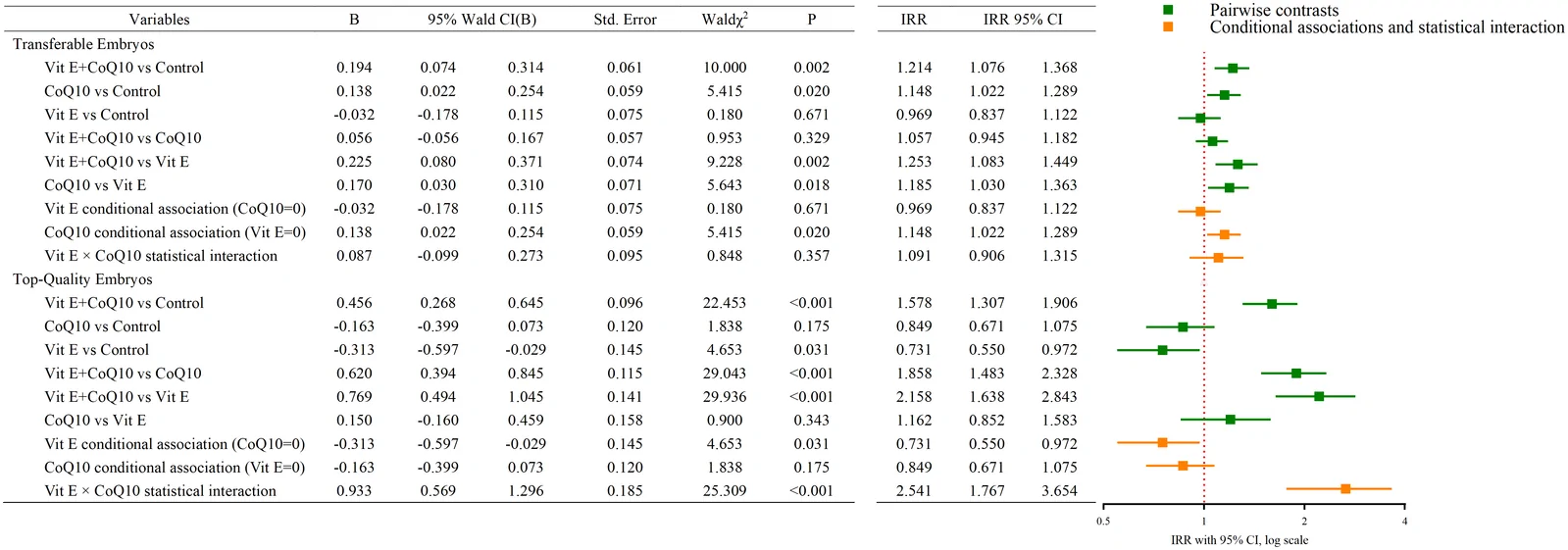
Generalized linear regression analysis of the associations between treatment regimens and embryological outcomes. Pairwise contrasts and the conditional main associations and statistical interaction of vitamin E and coenzyme Q10 with the numbers of transferable and top-quality embryos are presented. The models were adjusted for female age, partner age, body mass index, infertility duration, primary infertility status, basal FSH, AFC, AMH, basal estradiol, GnRH antagonist protocol use, total gonadotropin dosage, duration of gonadotropin stimulation, fertilization method, and treatment cycle initiation year. Adjusted estimates are expressed as IRRs with 95% CIs; the dashed line indicates an IRR of 1.

Model diagnostics did not indicate overdispersion. For the number of transferable embryos, the Pearson χ²/df and deviance/df values were 0.503 and 0.654, respectively; the corresponding values for the number of top-quality embryos were 0.814 and 0.897. The observed proportions of zero counts were 23.2% and 57.3%, compared with model-expected proportions of 37.5% and 60.5%, respectively, providing no evidence of excess zeros. In sensitivity analyses, the zero-inflated Poisson models converged, estimated negligible zero-inflation probabilities, and yielded virtually identical group-specific predicted counts. The Akaike information criterion values were slightly higher for the zero-inflated Poisson models than for the corresponding Poisson models (2234.3 vs 2232.3 for transferable embryos and 1740.7 vs 1738.7 for top-quality embryos). These findings supported the adequacy of the primary Poisson models.

The adjusted marginal mean counts and their 95% CIs for the four supplementation groups are presented in **Figure 5**. For transferable embryos, the adjusted predicted counts were 0.910 (95% CI 0.830–0.990) in the Control group, 1.104 (95% CI 1.013–1.196) in the Vit E+CoQ10 group, 1.045 (95% CI 0.966–1.123) in the CoQ10 group, and 0.882 (95% CI 0.777–0.986) in the Vit E group. For top-quality embryos, the corresponding adjusted predicted counts were 0.478 (95% CI 0.410–0.545), 0.754 (95% CI 0.661–0.847), 0.406 (95% CI 0.330–0.482), and 0.349 (95% CI 0.263–0.435), respectively.

**Figure 5:**
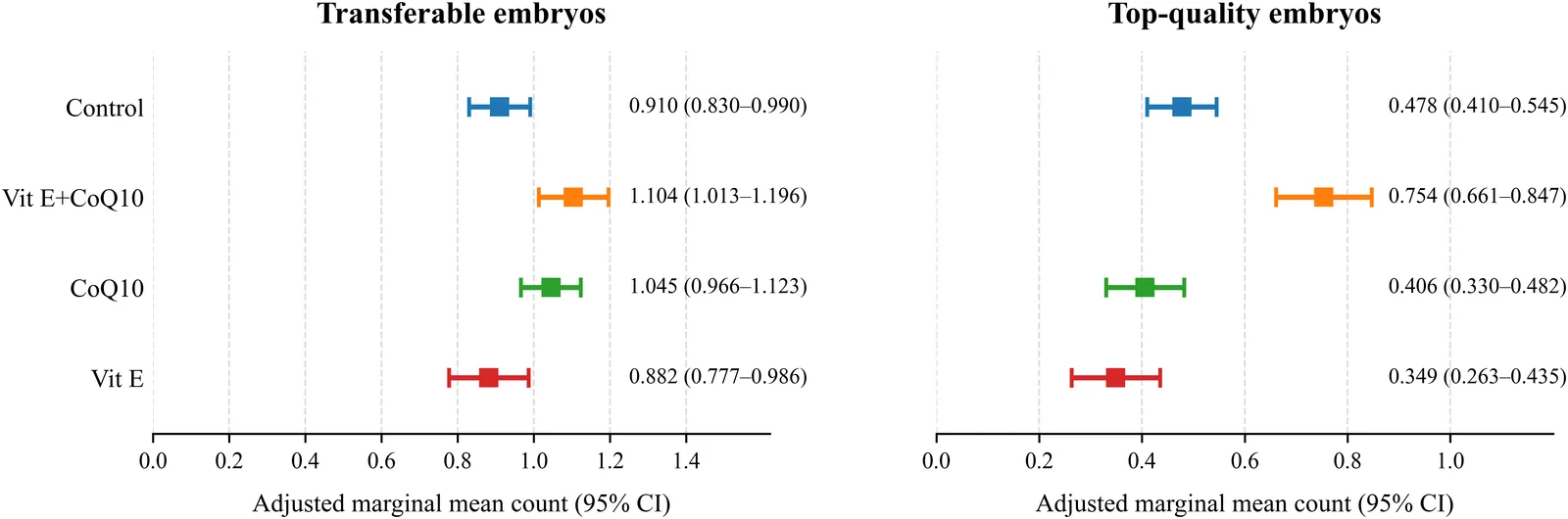
Adjusted marginal predicted counts of transferable and top-quality embryos by supplementation group. Points represent predicted counts, and error bars indicate 95% CIs. Estimates were derived from multivariable Poisson regression models with a log link and Huber–White robust standard errors, adjusted for the prespecified covariates described in the Statistical analysis section. Predictions were averaged over the observed covariate distribution.

## Discussion

The baseline characteristics of the patients with DOR included in this study did not differ significantly across groups, and differences in treatment outcomes following supplementation with Vit E and/or CoQ10 were primarily observed in the numbers of transferable embryos and top-quality embryos. After adjustment for the specified covariates and treatment cycle initiation year, with respect to the number of transferable embryos, both the Vit E+CoQ10 group and the CoQ10-alone group had significantly higher expected counts than the Control and Vit E-alone groups, whereas no statistically significant difference was observed between the combination and CoQ10-alone groups. Together with the nonsignificant Vit E-by-CoQ10 interaction, these findings suggest that the observed benefit in the number of transferable embryos may have been driven predominantly by the association with CoQ10 use. In contrast, a significant interaction was observed for the number of top-quality embryos. Notably, when CoQ10 was absent, Vit E use was associated with a lower expected number of top-quality embryos than no supplementation (IRR = 0.731, 95% CI: 0.550–0.972, P = 0.031). This estimate represents the conditional association of Vit E specifically when CoQ10 was absent and should not be interpreted as the overall association of Vit E across both CoQ10 exposure levels. Given the retrospective observational design and the possibility of residual confounding and treatment-selection differences, this finding should also not be interpreted as evidence that Vit E itself adversely affects embryo quality. The expected number of top-quality embryos in the Vit E+CoQ10 group was 57.8%, 85.8%, and 115.8% higher than that in the Control, CoQ10-alone, and Vit E-alone groups, respectively. Moreover, the interaction term between Vit E and CoQ10 was statistically significant (IRR = 2.541, 95% CI 1.767–3.654, *P* < 0.001), indicating a significant positive statistical interaction between their combined use and the number of top-quality embryos. This finding represents a positive statistical interaction on the multiplicative scale and, in the absence of direct mechanistic evidence, should not be interpreted as demonstrating biological or pharmacological synergy between Vit E and CoQ10. It should also be emphasized that supplementation with Vit E and/or CoQ10 was not associated with significant improvements in the clinical pregnancy, live birth, or early miscarriage rates among patients with DOR, suggesting that the observed improvements in embryological outcomes did not translate into clear benefits in clinical pregnancy outcomes.

Existing evidence suggests that CoQ10 may improve the ovarian response to controlled ovarian stimulation in women with DOR or poor ovarian response, as reflected by increases in the numbers of retrieved oocytes, mature oocytes, fertilized oocytes, and top-quality embryos, as well as reductions in gonadotropin requirements and cycle cancellation rates (4, 10). Some meta-analyses have further suggested that CoQ10 may increase the clinical pregnancy rate; however, no consistent evidence has demonstrated an improvement in the live birth rate or other pregnancy outcomes (11). These findings are generally consistent with the present observation that CoQ10 was associated primarily with improved embryological outcomes. Compared with CoQ10, clinical evidence regarding the effects of Vit E on ovarian function and reproductive outcomes in women with DOR remains limited. A randomized controlled trial reported that combined supplementation with selenium and Vit E might improve ovarian reserve in infertile women with occult premature ovarian insufficiency (OPOI), potentially by enhancing glutathione peroxidase activity and reducing reactive oxygen species–mediated follicular damage (9). However, the study had a small sample size and did not assess ovulation, pregnancy, live birth, or long-term reproductive outcomes. Therefore, its findings support only a potential improvement in surrogate markers of ovarian reserve and do not establish an increase in actual reproductive success. In addition, an experimental study of autologous intraperitoneal ovarian transplantation in rats found that Vit E attenuated oxidative damage and partially preserved the follicular reserve (12). Nevertheless, this was a small animal study in which Vit E was administered as a single intraperitoneal dose, and its findings cannot be directly extrapolated to oral supplementation in women. To date, clinical studies specifically evaluating combined Vit E and CoQ10 supplementation are lacking, as are direct comparisons among Vit E monotherapy, CoQ10 monotherapy, and their combination; consequently, the interaction between these two supplements has not been directly assessed. One study evaluating a multinutrient formulation containing both Vit E and CoQ10 found no significant improvement in the numbers of retrieved oocytes or top-quality embryos among patients undergoing IVF/ICSI-ET (13). This finding is not entirely consistent with the positive statistical interaction observed in the present study. However, that study had a relatively small sample size, and the multicomponent nature of the intervention precluded differentiation of the independent and combined associations of Vit E and CoQ10 with the study outcomes. By concurrently including a Vit E-alone group, a CoQ10-alone group, and a combination group, the present study provides new observational evidence supporting a possible positive statistical interaction between Vit E and CoQ10. Thus, the absence of significant clinical benefits in our cohort is compatible with the inconsistent evidence regarding clinical pregnancy and the insufficient evidence for improved live birth following CoQ10 supplementation. Several factors may explain why the observed improvements in embryological outcomes did not translate into detectable clinical benefits. Embryo morphological quality is an intermediate outcome and does not fully reflect embryonic chromosomal status, implantation competence, or ultimate live birth potential (14). Pregnancy and live birth are also influenced by multiple downstream factors, including embryo transfer strategy, endometrial receptivity, male factors, and maternal conditions (15). In addition, the number of live birth events is generally lower than the number of embryological events, and this study may not have had sufficient statistical power to detect relatively modest clinical effects.

The aforementioned effects of CoQ10 may primarily be attributed to its dual role in improving mitochondrial energy metabolism and enhancing antioxidant defense. CoQ10 is a lipid-soluble electron carrier in the mitochondrial electron transport chain, participating in electron transfer from complexes I/II to complex III and thereby supporting oxidative phosphorylation and adenosine triphosphate (ATP) production (16). Oocyte maturation, spindle formation, chromosome segregation, fertilization, and early embryonic cleavage all rely heavily on sufficient mitochondrial ATP supply (17). Therefore, CoQ10 may improve oocyte developmental competence by enhancing mitochondrial function and energy availability. The follicular microenvironment in patients with DOR may be characterized by increased oxidative stress (18). Excessive reactive oxygen species (ROS) can damage DNA, membrane lipids, mitochondria, and the meiotic spindle apparatus, thereby impairing oocyte maturation, fertilization, and early embryonic development (19). The reduced form of CoQ10, ubiquinol, has antioxidant properties and can scavenge free radicals, inhibit lipid peroxidation, and buffer oxidative stress induced by excessive ROS. Through these mechanisms, CoQ10 may help preserve mitochondrial function and cellular structural integrity required for oocyte competence and early embryonic development. The major active form of Vit E is α-tocopherol, a lipid-soluble chain-breaking antioxidant that is mainly distributed in cell membranes, mitochondrial membranes, and the lipid-rich environment of follicular fluid (20, 21). Its principal function is to interrupt lipid peroxidation chain reactions and stabilize membrane structures. In oocytes, Vit E may indirectly protect mitochondrial function, DNA stability, and spindle integrity by reducing ROS-related lipid peroxidation and oxidative damage, thereby contributing to the maintenance of oocyte quality (22). However, compared with CoQ10, the clinical evidence supporting Vit E alone for improving the number of retrieved oocytes, the number of top-quality embryos, or live birth rates in IVF/ICSI-ET remains limited. Another relatively well-studied area of Vit E in reproductive medicine is the endometrial environment. Some studies have shown that supplementation with Vit E at 400 IU/day is associated with increased endometrial thickness and improved oxidative stress markers, with certain effects observed particularly in populations with unexplained infertility, ovulation induction cycles, intrauterine insemination, or recurrent implantation failure (23). The potential mechanisms may include antioxidant effects, improvement of the endometrial microenvironment, and modulation of vascular-related factors. Nevertheless, current evidence remains insufficient to conclude that Vit E can consistently improve pregnancy or live birth rates in IVF/ICSI-ET. Therefore, Vit E is more appropriately described as a potential adjunctive antioxidant intervention. However, the current evidence regarding the effects of CoQ10 and Vit E on IVF/ICSI-ET outcomes has several limitations. Although the proposed mechanisms are biologically plausible, much of the evidence is derived from basic research, animal studies, *in vitro* experiments, or small clinical studies. Moreover, existing studies are heterogeneous in terms of patient populations, dosage regimens, duration of supplementation, and outcome measures. Current evidence more strongly supports their potential effects on intermediate outcomes, such as oxidative stress status, ovarian response, embryological parameters, or endometrial thickness, whereas consistent evidence for improvements in clinical pregnancy and live birth rates remains lacking. Accordingly, these agents should be regarded as potential adjunctive supportive interventions rather than definitive treatments for improving fertility outcomes.

The strengths of this study include its relatively large overall sample size, the inclusion of both monotherapy and combination therapy groups, and the use of main-effect and interaction models to distinguish the separate associations of CoQ10 and Vit E from their joint association. Nevertheless, several limitations should be acknowledged. First, supplementation was not randomly assigned, and treatment allocation may have been influenced by clinical characteristics, patient preferences, physician practices, or other unmeasured factors. Therefore, residual confounding caused by lifestyle factors, dietary antioxidant intake, adherence to supplementation, male partner factors, and other unmeasured variables cannot be completely excluded. In addition, cycles that were cancelled or interrupted before embryological outcomes could be adequately evaluated were excluded from the primary analyses. Although cancellation rates did not differ significantly among the four supplementation groups, exclusion based on a post-treatment event may still have introduced selection bias, particularly if the reasons for cancellation were associated with ovarian response or treatment exposure. Moreover, some cancelled cycles did not complete the planned 2-month supplementation regimen; therefore, the group-specific cancellation analysis was based on the documented supplementation category and should be interpreted cautiously. Second, baseline Vit E, CoQ10, or oxidative stress levels were not measured, making it impossible to determine whether the observed associations were concentrated among patients with nutritional insufficiency or higher oxidative stress. Third, embryo morphological grading may be subject to interobserver variability, and the lack of data on embryonic euploidy, blastocyst formation, and cumulative live birth outcomes limited the assessment of true embryonic developmental potential and ultimate clinical value. Finally, since no single primary embryological outcome was prespecified and multiple outcomes and pairwise comparisons were assessed, these findings should be considered exploratory and require confirmation in prospective studies.

## Conclusion

In summary, CoQ10 supplementation was associated with an increased number of transferable embryos, while the combined use of Vit E and CoQ10 was associated with a higher number of top-quality embryos, and a positive statistical interaction between Vit E and CoQ10 was observed. However, this embryological advantage did not translate into significant improvements in pregnancy or live birth rates. Future multicenter randomized controlled trials with standardized dosage regimens and supplementation durations are warranted. Such studies should incorporate biomarkers of oxidative stress and mitochondrial function, embryonic euploidy, blastocyst formation rate, and cumulative live birth rate to further clarify the target population, underlying mechanisms, and clinical value of combined supplementation. Therefore, the present findings should be interpreted as embryological evidence supporting further randomized trials, rather than as a basis for directly recommending Vit E and CoQ10 as routine adjunctive treatments in IVF/ICSI-ET.

## Data Availability

The raw data supporting the conclusions of this article will be made available by the authors, without undue reservation.
